# Green Tea Polyphenol Epigallocatechin Gallate Interactions with Copper-Serum Albumin

**DOI:** 10.3390/molecules30020320

**Published:** 2025-01-15

**Authors:** Meiling Fu, Liangliang Zhang, Rick Killeen, Kenneth E. Onugwu, Robert M. McCarrick, Ann E. Hagerman

**Affiliations:** 1 Department of Chemistry & Biochemistry, Miami University, Oxford, OH 45056, USA; fum6@miamioh.edu (M.F.); kennethonugwu@yahoo.com (K.E.O.); rob.mccarrick@miamioh.edu (R.M.M.); 2 Institute of Advanced Carbon Conversion Technology, Huaqiao University, Xiamen 361021, China; zhangll@hqu.edu.cn; 3 Department of Anesthesia and Perioperative Medicine, Medical University of South Carolina, Charleston, SC 29425, USA; killeen@musc.edu

**Keywords:** polyphenol, metalloprotein, antioxidant, serum albumin, copper, green tea

## Abstract

Epigallocatechin gallate (EGCg), an abundant phytochemical in green tea, is an antioxidant that also binds proteins and complex metals. After gastrointestinal absorption, EGCg binds to serum albumin in the hydrophobic pocket between domains IIA and IIIA and overlaps with the Sudlow I site. Serum albumin also has two metal binding sites, a high-affinity N-terminal site (NTS) site that selectively binds Cu(II), and a low-affinity, less selective multi-metal binding site (MBS). We proposed to determine whether EGCg binds or reacts with Cu(II)-serum albumin using fluorescence, UV–Visible and electron paramagnetic resonance (EPR) spectroscopy. Our results suggest that when serum albumin is loaded with Cu(II) in both sites, EGCg binds to the MBS-Cu(II) and reduces the copper to Cu(I). EGCg does not bind to or react with Cu(II) in the high-affinity NTS site. Potential consequences include changes in copper homeostasis and damage from pro-oxidative Fenton reactions.

## 1. Introduction

Polyphenols are naturally occurring compounds widely found in plant-based foods and beverages [1]. The bioactivities of polyphenols include their tendency to tightly bind protein, to act as antioxidants and to bind metals. Protein binding, particularly by the high molecular weight polyphenols known as tannins, can contribute to protein malnutrition among malnourished populations [2]. Furthermore, polyphenol-rich foods can diminish the bioavailability of non-heme iron, leading to anemia [3]. Dietary polyphenols are also reputed to have positive effects on human health as a consequence of their antioxidant activity [4]. Many recent studies have focused on the ability of polyphenols to quench radicals, such as the reactive oxygen species that are associated with diseases including cardiovascular disease, carcinogenesis, and neurodegenerative diseases [5]. Redox activities of polyphenols with metals such as iron or copper have been relatively neglected despite the biological importance of these metals and the well-known ability of polyphenols to chelate metals [3,6].

**Figure 1:**
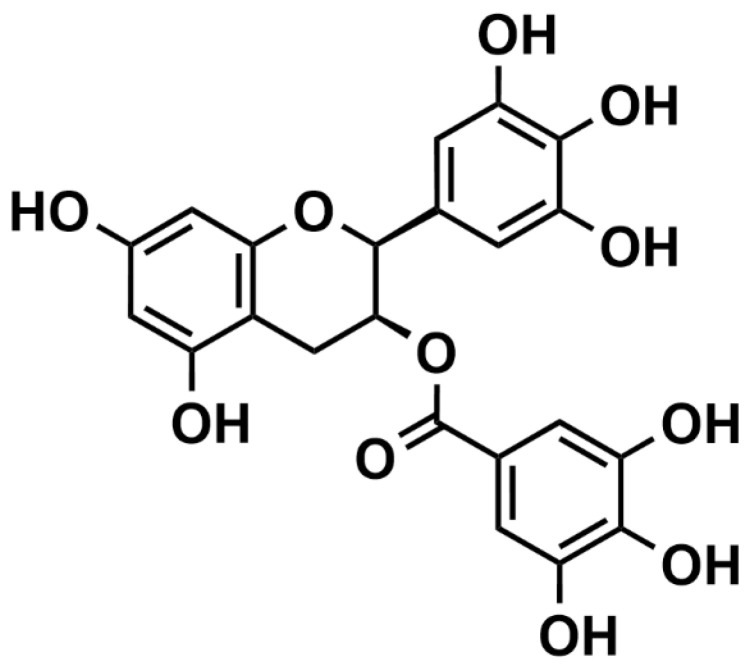
Structural formula of epigallocatechin gallate (EGCg).

An important dietary polyphenol is epigallocatechin gallate (EGCg) (Figure 1), the primary polyphenol in green tea, with ingestion of as high as 20–50 mg/day in tea-drinkers [7]. In tea, EGCg can aggregate milk proteins to form a turbid suspension, but the EGCg concentration must exceed that of the protein around fivefold (by mass) before precipitation occurs [8]. In the digestive tract, EGCg quickly degrades to oxidation products unless it is stabilized by protein or other dietary components [9]. The small amounts of EGCg absorbed from the gastrointestinal tract are likely to be transported by serum albumin, with unknown fate and effects [7,8]. EGCg does chelate Cu(II) and reduces Cu(II) to Cu(I) in vitro [10,11,12], but the relationship between EGCg and copper in vivo is poorly known.

Copper is a micronutrient with essential roles in redox enzymes, including cytochrome c oxidase and superoxide dismutase, but excess copper may contribute to pathologies such as cancer or Alzheimer’s disease [13]. The human body contains approximately 100 mg of copper, with a recommended daily intake of 0.9 mg [14,15]. Copper uptake and excretion are tightly regulated by biological systems [16]. Under normal physiological conditions, most of the copper in plasma is bound to ceruloplasmin (85–95%), but diseases can disrupt the pool. For example, in Wilson’s disease, an abnormally low concentration of copper is ceruloplasmin-bound and unusually large amounts are transported by serum albumin [17,18].

Serum albumin is the most abundant protein in human and animal blood. It is well known for its ability to transport various ligands, including polyphenols, metal ions, and certain drugs [19]. Bovine serum albumin (BSA), which is a close homolog to human serum albumin, contains 583 amino acids consisting of three homologous all-α domains, organized into a heart-shaped structure [20]. Serum albumin has two major Cu(II) binding sites [21]. The affinity constant of the N-terminal binding site (NTS) for Cu(II) is in the picomolar range [22]. The multi-metal binding site (MBS), which is found at the interface between domains I and II of the protein, binds various metals such as Cu(II), Zn(II), Ni(II), or Co(II) with relatively low affinity in the nanomolar range [23]. The NTS site comprises the strong field ligands N-terminal amine, the amide bonds at Asp-1 and Ala-2 (human) or Thr-2 (bovine), and the imidazole of His-3 [22] arranged in square planar geometry around the Cu(II). The weak field ligands at the multi-metal binding site (MBS) yield a pentacoordinate geometry with N and O donors from His-67, Asn-99, His-246 and Asp-248 in the equatorial positions [24]. Water in the axial site points towards the outside of the protein and is accessible to other ligands [25].

In addition to binding metals, serum albumin binds many organic ligands, including drugs and dietary polyphenols [26]. Two well-characterized drug binding sites, the Sudlow site I and Sudlow site II, are found in the hydrophobic cavities at subdomains IIA and IIIA, respectively [27]. Fluorescence spectroscopy and molecular modeling were used to demonstrate that EGCg binds to a hydrophobic pocket between domain IIA and IIIA, and that overlaps the Sudlow I site [28]. Additional studies used ITC and NMR to confirm both strong and weak EGCg binding sites on serum albumin [29].

The diverse binding sites on serum albumin permit the formation of multi-ligand complexes that can exhibit unique functional features such as drug bioavailability because of ligand competition for sites or ligand-induced conformational modulation [30]. The importance of metallo-organic compounds as pharmacological agents has stimulated interest in ternary interactions between serum albumin and metal–organic complexes such as cisplatin [31]. Ternary complexes between proteins and polyphenol–metal complexes have received limited attention [32,33], although the binding of pre-formed Cu(II)-phenolic complexes to serum albumin have been characterized in vitro and in silico [34,35,36]. Cu(II)-EGCg binds in the same hydrophobic site but has a higher affinity than uncomplexed EGCg [28,34]. There was no indication of electron transfer from the polyphenol to the Cu(II) in the presence or absence of protein and no indication that the copper-EGCg interacted with the protein at either the NTS or the MBS binding site.

The aim of our study was to extend our knowledge of ternary complexes between serum albumin, Cu(II) and EGCg using serum albumin that was loaded with copper at either the NTS site or the NTS and MBS site. We proposed that EGCg might interact preferentially with the MBS-bound copper, since the ligands at that site are weaker than those at the high-affinity copper-binding sites (NTSs). We explored possible electron transfer between the protein-bound copper and EGCg by using electron paramagnetic resonance spectroscopy (EPR) to characterize the copper, and we examined the polyphenol binding site and copper ligands with fluorescence and UV–Visible spectroscopy. Our study expands our understanding of the diverse bioactivities of polyphenols and characterizes a novel ternary complex between serum albumin, copper, and green tea polyphenol EGCg.

## 2. Results

### 2.1. Interactions Between Cu(II) and BSA

**Figure 2:**
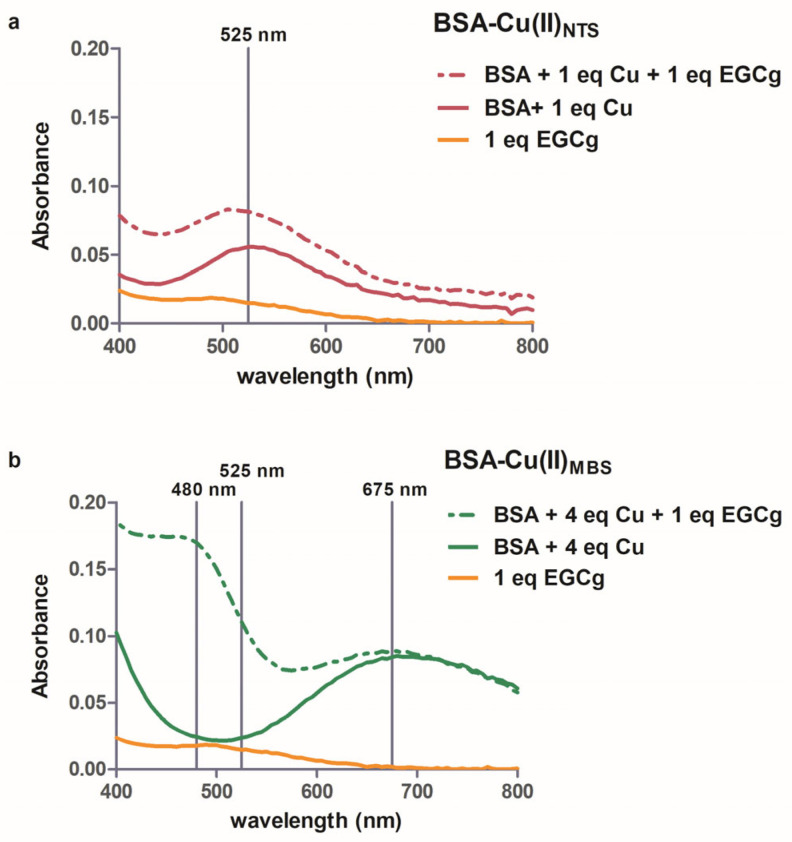
Visible spectra of 1 mM BSA-Cu(II)_NTS_ (**a**) or 1 mM BSA-Cu(II)_MBS_ (**b**). Spectra without EGCg (solid line) and with one equivalent of EGCG (dashed line) are shown. To obtain the spectra in (**a**), the absorbance of BSA was subtracted from the data. For (**b**), the absorbance of BSA-Cu(II)_NTS_ was subtracted from the data. The orange line is the spectrum of 1 mM EGCg. The vertical lines indicate 525 nm (**a**) and 480 nm, 525 nm, and 675 nm (**b**).

BSA has two binding sites for copper, the high-affinity NTS (N-terminal site) and lower affinity, less specific MBS [23]. The visible spectra of Cu(II) bound to each site are distinct because of the unique ligands at each site [37]. When copper is added to serum albumin, the first equivalent binds to the NTS, where it interacts with four strong field N ligands, yielding a complex with λ_max_ 525 nm (Figure 2a, solid red line). When copper is added in excess of the first equivalent, it binds to the lower affinity MBS, which has a broad λ_max_ between 650–700 nm [37], shown by the difference spectrum for the protein with four equivalents of copper (Figure 2b, solid green line). Similar spectra were obtained with either MOPS or phosphate buffer.

**Figure 3:**
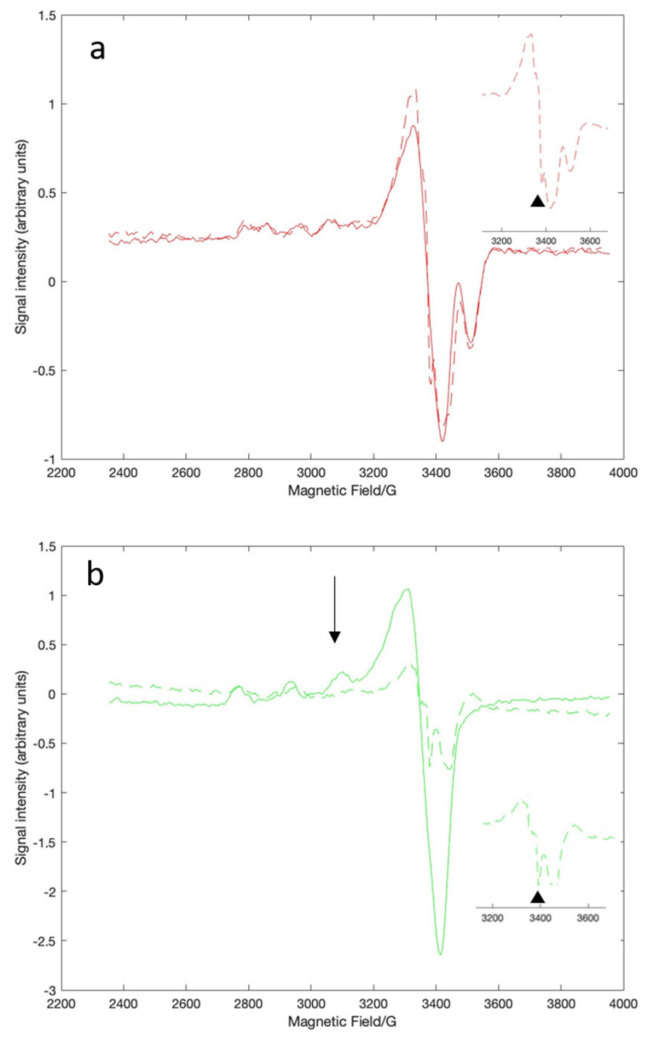
X-band EPR spectra of BSA-Cu(II)_NTS_ (**a**) or BSA-Cu(II)_MBS_ (**b**). Spectra without EGCg (solid line) and with one equivalent of EGCG (dashed line) are shown. The spectrum of BSA-Cu(II)_NTS_ (**a**) was obtained using the histidine-Cu(II) complex and ultrafiltering the sample to remove excess Cu(II). The spectrum of BSA-Cu(II)_MBS_ (**b**) is the difference spectrum obtained by subtracting the BSA-Cu(II)_NTS_ signal. The EGCg radical signal is indicated by the black triangles in the insets. The unique BSA-Cu(II)_MBS_ signal is indicated by the black arrow. All samples were in MOPS buffer at pH = 7.4.

EPR spectroscopy was used to look more specifically at the copper-serum albumin interactions. The relative affinities for serum albumin for Cu(II) are NTS > histidine > MBS [22], so introducing Cu(II) as the histidine complex ensured that we could obtain the EPR spectrum of BSA-Cu(II)_NTS_ without interference from copper in the MBS (Figure 3a, solid red line). We prepared BSA-Cu(II)_NTS+MBS_ by loading the protein with three equivalents of CuCl_2_. The difference spectrum obtained by subtracting the spectrum of BSA-Cu(II)_NTS_ from the spectrum of BSA-Cu(II)_NTS+MBS_ represents BSA-Cu(II)_MBS_ (Figure 3b, solid green line). Simulation of the experimental spectra revealed the quantitative features that differentiate the spectra of Cu(II)_MBS_ from Cu(II)_NTS_, including their specific g tensor and hyperfine coupling values (Table 1, Figure S1). The values are consistent with previous reports that the ligand geometry for NTS is square planar while MBS is square pyramidal [22,23]. These assignments are consistent with the Peisach–Blumberg correlation diagram for 4N ligands for the NTS site and for 2N, 2O ligands for the MBS site (Figure S2) [38]. The fifth ligand for the MBS site is water, with other external ligands such as buffers modulating the coordination sphere [24].

Our data confirmed that Cu(II) is a weak quenching agent for the intrinsic fluorescence of BSA (Figure S3) [39]. The spectra indicated that serum albumin was not denatured by Cu(II) even when large excesses (three equivalents) of the metal were added to the protein. Reports that copper facilitates denaturation and aggregation used elevated temperatures to prompt fibrillation of the protein and are not relevant to our room temperature conditions or to physiological conditions [40].

### 2.2. Interactions Between EGCg and BSA

**Figure 4:**
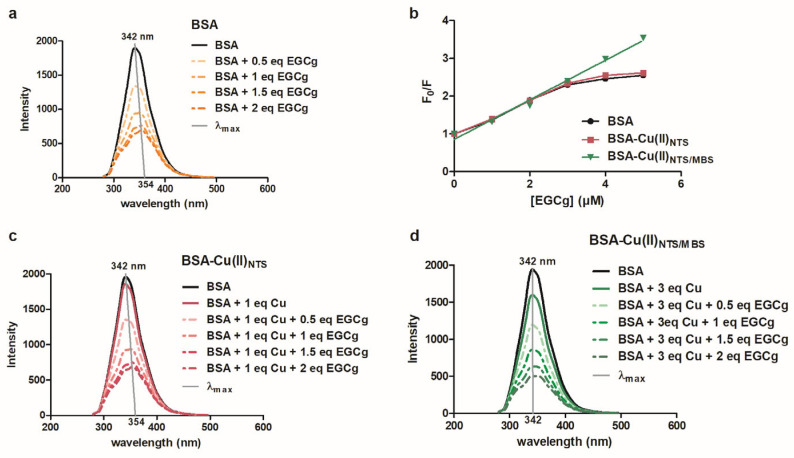
Fluorescence emission of BSA or BSA-Cu titrated with EGCg. BSA (**a**), BSA-Cu(II)_NTS_ (**c**), or BSA-Cu(II)_NTS/MBS_ (**d**) was titrated with EGCg. The λ_max_ for each curve is indicated by a gray line. The λ_max_ for BSA or BSA-Cu is 342 nm. The λ_max_ for BSA or BSA-Cu(II)_NTS_ is red-shifted to 354 nm by the addition of EGCg. The Stern–Volmer plots (**b**) are curved for BSA or BSA-Cu(II)_NTS_ and yield a K_a_ = 0.32 μM^−1^ using the modified Stern–Volmer analysis. The linear SV plot for EGCg binding to BSA-Cu(II)_NTS/MBS_ had an equation of F/F_o_ = 0.52 × [EGCg] + 0.85 (r^2^ = 0.99), yielding a K_SV_ = 0.52 μM^−1^. The BSA concentration was 2 mM and the CuCl_2_ concentration was 2 mM (**c**) or 6 mM (**d**).

EGCg interactions with serum albumin have been examined using a wide range of techniques, including fluorescence quenching spectroscopy, isothermal titration calorimetry, CD spectroscopy, NMR, FRET, and MALDI-TOF-MS [28,29,41,42]. Our lab [28] showed that EGCg quenches the fluorescence of Trp 213 with a red shift (12.5 nm) when it binds to a high-affinity site for EGCg on serum albumin in the hydrophobic pocket between domains IIA and IIIA and overlapping with the Sudlow I site (Figure 4a). Similar to earlier reports [28], the Stern–Volmer plot is curved for EGCg binding to BSA, with a K_a_ = 0.32 μM^−1^ using the modified Stern–Volmer analysis (Figure 4b). EGCg binding to the Sudlow II site and other weak, nonspecific binding is not detected by fluorescence spectroscopy [29].

We did not obtain any information about the interaction between EGCg and BSA from UV–Visible spectra of the complexes. The spectra had no unique features beyond the sum of the spectra of the individual species. There were no radical signals in the EPR spectra of metal-free mixtures of EGCg and serum albumin, so no structural information could be derived. In previous studies, oxygen-dependent formation of EGCg radicals could be detected in EGCg-BSA mixtures by adding zinc to stabilize EGCg radical species [43], but in that study, specific structural details of the interaction between the protein and the phenolic radical could not be established from the EPR spectra.

### 2.3. Interactions Between EGCg and Cu(II)

Polyphenols typically have one or more *ortho* diphenolic moieties (Figure 1), making them ligands of metals such as Fe(III), Al(III), Cu(II), and Zn(II) [44]. Furthermore, the low reduction potential of polyphenols ensures that they can reduce metals such as Fe(III) and Cu(II) [45]. Although fluorescence did not provide any information about EGCg–copper interactions, we were able to characterize complex formation and redox chemistry for EGCg and Cu(II) with UV–Visible and EPR spectroscopy.

**Figure 5:**
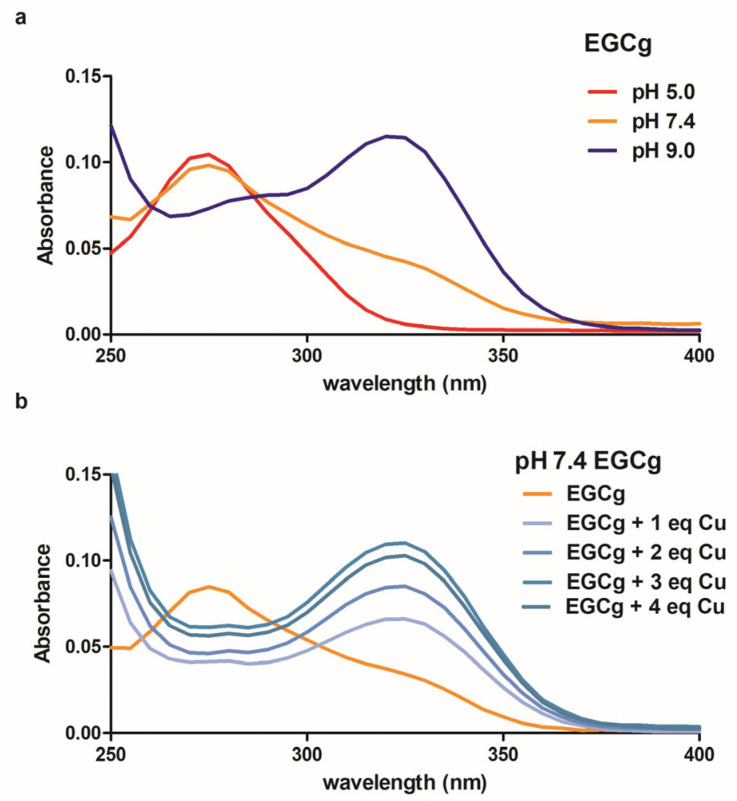
UV–Visible spectra of 10 μM EGCg at acid, neutral and basic pH (**a**). Spectra of the EGCg in pH 7.4 phosphate buffered solution with added CuCl_2_ (**b**). To obtain the spectra in (**b**), the absorbance of CuCl_2_ was subtracted from the data.

UV–Visible spectroscopy allowed us to relate formation of the EGCg-Cu(II) complex to deprotonation of the phenolic hydrogens, similar to previous studies of EGCg and other polyphenols with several metals [6,46]. When an aqueous solution of EGCg is titrated with base, the characteristic 275 nm absorbance peak is replaced by a peak at 325 nm as the B-ring ortho diphenol groups are deprotonated (Figure 5a) with a first pKa of approximately 8.0. The deprotonation does not cleanly proceed through an isosbestic point probably due to the formation and subsequent rearrangement of EGCg oxidation products to complex species [47]. We added Cu(II) to a pH 7.4 buffered solution of EGCg and noted the concentration-dependent loss of the 275 nm absorbance peak and formation of the 325 nm peak due to metal-induced deprotonation (Figure 5b). The spectrum of EGCg plus Cu(II) was indistinguishable from the spectrum of deprotonated EGCg. There was no evidence for lower energy-absorbing charge transfer or d-d complexes between EGCg and Cu(II) [48,49]. Furthermore, during the short time of the UV–Visible experiment, no oxidative browning was observed although eventually Cu(II) promotes oxidation of the EGCg [50].

The EPR spectra of mixtures of Cu with up to five equivalents of EGCg spectrum at pH 7.4 confirmed that the complexes formed at pH 7.4 in MOPS buffer were Cu(II)-EGCg_2_ [12]. As noted in other studies, EGCg semiquinone radicals were not detected in the spectrum of the copper complex, and there was no evidence for the reduction of Cu(II) by the polyphenol [12,51].

### 2.4. Interactions Between BSA, Cu(II) and EGCg (NTS Site)

Visible spectroscopy showed that the characteristic BSA-Cu(II)_NTS_ peak at 525 nm was not changed by the addition of one equivalent of EGCg (Figure 2a, dashed red line), suggesting that the Cu(II) bound to NTS does not react with EGCg. Similar spectra with λ_max_ 525 nm were obtained with up to three equivalents of EGCg added to the BSA-Cu(II)_NTS_, although we did note an increased intensity of the peak at 525 nm due to the absorbance of the EGCg solution at pH 7.4 (Figure 2a, dashed red line). In a similar study, ascorbic acid was added to BSA-Cu(II), and the absorbance at 525 nm decreased as the ascorbic acid reduced the Cu(II) to Cu(I) [52]. The polar nature of ascorbic acid promotes its reactivity with Cu(II) bound at the surface-localized NTS site on the protein, but the polyphenol EGCg binds to the hydrophobic pocket near the Sudlow I site on the protein [28,29] and is sequestered away from the NTS site. EGCg is far less polar than ascorbic acid, as shown by their octanol-water partition coefficients (partition coefficients XlogP 1.2 and −1.6, respectively, PubChem database).

The EPR data were consistent with a model in which EGCg does not interact with BSA-Cu(II)_NTS_. The addition of as much as 2.5 equivalents of EGCg did not change the major features of the EPR spectrum of BSA-Cu(II)_NTS_ (Figure 3a, dashed red line). The overall similarity of the spectra of the NTS site-bound Cu(II) in the absence and presence of the polyphenol suggests that the coordination state of the Cu is not affected by the addition of EGCg. However, a radical signal is visible as a very small new sharp peak in the EPR spectrum of BSA-Cu(II)_NTS_ (Figure 3a, inset), indicating that some electron transfer from EGCg to Cu(II) has taken place. EPR is very sensitive and reveals small amounts of radical that are undetectable in the weak visble bands characteristic of these complexes.

Additional supporting evidence for limited interaction between EGCg and NTS-Cu(II) was provided by fluorescence spectroscopy. Fluorescence quenching by EGCg was similar for BSA or BSA-Cu(II)_NTS_ (Figure 4a,c). For both forms of the protein, quenching was accompanied by a 12 nm red shift and curved Stern–Volmer plot (K_a_ = 0.32 μM^−1^) (Figure 4b). The similar nature of the fluorescence quenching for BSA and BSA-Cu(II)_NTS_ verifies that EGCg binds to the hydrophobic pocket of BSA-Cu(II)_NTS_ without interacting with the Cu(II) in the NTS site.

### 2.5. Interactions Between BSA, Cu(II) and EGCg (MBS Site)

The interactions between BSA-Cu(II)_NTS_ and EGCg could be explored in isolation from the MBS site by using Cu(II)-His_2_ to deliver the metal to the protein [22]. However, the interactions between EGCg and copper in the MBS site could not be examined independently. The low-affinity MBS site is occupied by Cu(II) only after the NBS site is filled. Nonetheless, we were able to assess the MBS site interactions indirectly.

Visible spectroscopy indicated that EGCg interacts with Cu(II) in the MBS site of serum albumin. The distinctive peak with λ_max_ 675 nm is unperturbed by the addition of EGCg to the BSA-Cu(II)_MBS_ (Figure 2b, dashed green line). However, the addition of EGCg to the copper-saturated protein is accompanied by the appearance of a new spectral feature that has a maximum absorbance at 480 nm (Figure 2b, dashed green line). Phenolate–copper complexes characteristically exhibit a strong absorbance between 410–545 nm due to phenolate-to-copper charge transfer [49]. Appearance of this new band in the BSA-Cu(II)_MBS_-EGCg spectrum suggests that EGCg is a ligand of the MBS-bound Cu(II). It is likely that EGCg displaces the weakly bound axial water in the MBS binding site [25].

EPR spectroscopy added further insight into the interactions between EGCg and the Cu(II) in the MBS site. The unique signal (Figure 3b, black arrow) associated with MBS-bound Cu(II) decreased in intensity as increasing amounts of EGCg were added to the copper-loaded protein with complete loss of signal at 2.5 equivalents of EGCg (Figure 3b, dashed green line). Loss of the MBS feature indicates that the Cu(II) coordination in the MBS site is affected by EGCg, which is consistent with the visible spectra. The overall intensity of the EPR spectrum decreased with increasing EGCg (Figure 3b, dashed green line), which is consistent with the reduction of Cu(II) to Cu(I). Cu(I) is not visible in EPR. Furthermore, a strong EGCg radical signal appeared, indicating electron transfer from the polyphenol to the copper (Figure 3b, inset). The EGCg radical signal is more intense than the weak radical signal noted with BSA-Cu(II)_NTS_ (Figure 3a, inset).

Fluorescence quenching data provide additional support for a model in which EGCg interacts with copper-loaded serum albumin at the Cu(II)_MBS_ binding site. EGCg quenches the intrinsic fluorescence of BSA-Cu(II)_MBS_ in a linear fashion with little change in the emission spectrum λ_max_ (Figure 4b,d), yielding a K_SV_ = 0.52 μM^−1^. The linearity of the Stern–Volmer plot shows that when the NTS and MBS sites on BSA are filled with Cu(II), both the hydrophobic pocket Trp213 and the surface-localized Trp134 are fully accessible to EGCg (Figure 4b). There is little spectral shift in the quenched spectrum because EGCg interacts with buried and surface Trp, with limited overall effect on the polar environment near those resides.

## 3. Discussion

Our data extend our knowledge of ternary complexes between serum albumin, Cu(II) and EGCg by showing that the BSA-Cu(II)-EGCg interaction depends on the extent of copper loading of the protein. When our experimental conditions were designed with low Cu(II) levels, and all of the copper was in the NTS site of the BSA, Cu(II) had little effect on EGCg. Similar to previous reports, the EGCg does not affect the coordination or redox status of the NTS-bound copper [53]. When our experimental conditions were designed with higher copper levels so the protein has copper in both the NTS and the MBS site, EGCg participates in coordination of the copper at the MBS site. The role of EGCg may be similar to that of buffers such as Hepes, replacing the axial water ligand at the BSA-Cu(II)_MBS_ site [24]. Unlike the inert buffers, EGCg is a reactive ligand that reduces Cu(II) to Cu(I) with the formation of the EGCg radical. Oxidation by BSA-Cu(II)_MBS_ could reduce the lifetime of EGCg in the blood.

Several studies have suggested that extended lifetimes of polyphenols such as quercetin can be achieved by using proteins such as serum albumin to control reactions of the polyphenol with Cu(II) [54,55]. Our studies reveal more nuance, with the ratio of Cu(II) to BSA determining the fate of the EGCg. When the copper-to-protein ratio is low, the protein effectively protects EGCg by sequestering it near the Sudlow I site, away from the NTS-bound metal. If copper levels are higher, the excess copper binds to the MBS site and is accessible to EGCg. In addition to its binding site on the protein, the polyphenol binds to the Cu(II) in the MBS site. When EGCg binds to Cu(II), a redox reaction occurs, yielding EGCg radicals, Cu(I), and ROS. The lifetime of the EGCg is diminished by its interaction with copper at the MBS site.

Our experiments modeled typical physiological conditions by using low EGCg concentrations rather than the high levels of EGCg that are associated with hepatotoxicity [56]. EGCg toxicity is a consequence of oxidative decomposition reactions yielding phenolic radicals with the potential to directly damage biomolecules including protein, nucleic acids and other metabolites [1,57,58,59,60]. We found that EGCg oxidation was dependent on the Cu(II) levels. Contradictory to our findings, it has been reported that in vivo, the toxicity of EGCg is diminished by high levels of copper [61]. The authors reported that high doses of copper increased levels of blood ceruloplasmin [61], a protein that typically binds approximately 90% of serum Cu(II) [53]. Apparently, ceruloplasmin quenched the oxidative destruction of EGCg, but the specific details of ceruloplasmin-Cu(II)-EGCg interactions and molecular mechanisms for the diminished toxicity were not clearly established [61].

The poor bioavailability of EGCg acts in concert with its low stability to minimize its pharmacological potential [62]. Typical intakes of approximately 20 mg of EGCg per day lead to micromolar levels of EGCg in the blood [62,63], with the polyphenol mainly bound to proteins such as serum albumin. Low micromolar affinity constants were obtained for EGCg binding to BSA, BSA-Cu(II)_NTS_ or BSA-Cu(II)_MBS_, suggesting that the formation of EGCg-serum albumin complexes is independent of copper status. In contrast, pre-formed Cu(II)-flavonoid complexes can have a higher affinity for serum albumin compared to the uncomplexed flavonoid [64]. Further direct studies of the role of copper in dictating free vs. bound polyphenol under physiological conditions are warranted to better understand bioavailability.

There is a long history of interest in the health benefits of EGCg [65,66], leading to increased use of supplements to augment the natural dietary intake of EGCg [67]. Many of the claims for EGCg are associated with its antioxidant activity, but antioxidants have the potential to promote oxidative damage if their oxidized form is highly active. Our study highlights how proteins such as serum albumin can participate in the formation of pro-oxidative EGCg when Cu(II) is present in sufficient concentration. To fully harness the potential benefits but avoid the likely risks of dietary polyphenols such as EGCg, more detailed studies of physiologically relevant ternary complexes of protein–metal–polyphenol should be conducted.

## 4. Materials and Methods

### 4.1. Materials

EGCg was a gift from Lipton Tea Co. (London, UK). Fatty acid free BSA (PDB P02769) was from Sigma Chemicals (St. Louis, MO, USA). Comparison sequences for human serum albumin used PDB Q56G89. All other reagents were reagent grade or better.

### 4.2. UV–Visible Spectroscopy

All spectra were recorded in 1 cm quartz cuvettes on a Cary 60 UV–Visible spectrometer (Agilent, Santa Clara, CA, USA) between 200–800 nm with an appropriate buffer blank. A BSA stock solution (1.2 mM) was prepared in 200 mM sodium phosphate or 200 mM MOPs buffer at pH 7.4. Stock solutions (40 mM) of EGCg and CuCl_2_ were prepared in nanopore water. BSA was mixed with the desired amount of CuCl_2_ and diluted to 1.0 mL to yield a final concentration of 1 mM. Copper levels ranged from 0 equivalents to 4 equivalents of copper relative to BSA. The samples were mixed well and incubated at room temperature for 30 min before transferring to a 1 cm quartz microcuvette. EGCg was added in 0.5 molar equivalent steps to reach 2.5 equivalents of EGCg relative to BSA. After each addition, the sample was immediately mixed by inverting the cuvette and the spectrum was recorded.

To establish spectra of protonated and deprotonated EGCg, spectra were obtained for 10 µM samples of EGCg in 200 mM phosphate buffer at pH 5, 7 or 9. The solutions of EGCg in pH 7 buffer were titrated with up to 4 equivalents of Cu(II).

### 4.3. Fluorescence Spectroscopy

A stock of 2 µM BSA was prepared in 20 mM sodium phosphate buffer at pH 7.4. A stock of 10 mM EGCg was prepared in 50% methanol and diluted to 0.5 mM using nanopure water. CuCl_2_ solution (40 mM) was prepared in 0.1 M NaCl and diluted as needed to achieve the desired final reaction mixture. Mixtures were prepared containing 3.00 mL of 2 µM BSA plus up to 12 µL of Cu solution to yield up to 6 µM Cu (up to three equivalents of Cu relative to BSA). Each mixture was titrated with successive 6 µL aliquots of 0.5 mM EGCg to achieve 1 to 4 µM EGCg (0.5 to 2 equivalents of EGCg relative to BSA) in the reaction mixture. Samples were mixed by inversion and incubated 10 min before taking the spectra. At least three technical replicates were done for each titration.

Fluorescence spectra were collected with a PerkinElmer LS 55 Fluorescence Spectrometer (Waltham, MA, USA) with F1 firmware, R928 detector type and voltage 775 V. The excitation wavelength was set at 275 nm and the emission wavelength was recorded from 280 nm to 500 nm. The slit widths for excitation and emission were 15.0 nm and 4.0 nm, respectively. Fluorescence intensity at 275 nm was used after correcting for sample absorbance according to Equation (1).

(1) $$F_{cor}=F_{ori}\times 10^{A275}$$

*F_cor_* and *F_ori_* are the corrected and original fluorescence intensity, respectively. *A*275 is the absorbance of the sample at 275 nm, which was collected on an Agilent 8453 spectrophotometer using a titration procedure exactly like that used for the fluorescence analysis.

If all fluorophores in the system are equally accessible, fluorescence quenching is described by Stern–Volmer equation (Equation (2)), yielding a linear Stern–Volmer plot [68].

(2) F0F=1+KSV[Q]

*F*_0_ and *F* are the fluorescence intensities without and with the quencher, respectively. *Ks_V_* is the Stern–Volmer quenching constant. [*Q*] is the concentration of the quencher.

### 4.4. EPR Spectroscopy

EPR data were collected in MOPS, a buffer that quenches the EPR signal of Cu(II)-aquo complexes (Figure S4) [22]. The BSA stock solution (2.4 mM) was prepared in 200 mM MOPs buffer at pH 7.4. Stock solutions (40 mM) of EGCg and CuCl_2_ were prepared in nanopore water. MOPs containing 13.5% (*v*/*v*) glycerol was prepared by adding the desired amount of glycerol to 200 mM MOPs buffer. The solution of histidine-chelated copper (Cu(II)-His_2_) contained 40 mM CuCl_2_ dissolved in 80 mM L-Histidine-HCl.

Reaction mixtures containing 0.5 mM BSA, 10% glycerol, and up to three equivalents of copper relative to BSA were prepared by mixing BSA stock solution with glycerol-MOPs, adding the required amount of CuCl_2_ stock solution and then diluting to 1.0 mL with MOPs buffer. The desired amount of EGCg stock solution was added to BSA or the BSA–copper complex and the sample was transferred to a 4 mm EPR tube to take the EPR spectrum at 5 K within 4 h.

In order to obtain an EPR spectrum of NTS-Cu without interference from copper in the MBS site, three equivalents of Cu(II)-His_2_ stock solution was added to the BSA stock solution and incubated at room temperature for 10 min. The mixture was ultrafiltered (Millipore Amicon^®^ Ultra 0.5 with 30 kD cutoff) (Burlington, MA, USA) at 12,500× *g* for 15 min at 4 °C to remove excess Cu(II)-His_2_. The retentate was re-filtered three times with a small volume of MOPS buffer, and retrieved by inverting the insert and centrifuging at 1000× *g* for 2 min. The solution was diluted with MOPs buffer to the original volume. Complete removal of free Cu-His_2_ was confirmed by inductively coupled plasma mass spectrometry (ICP-MS) analysis of the final filtrate, which contained only 13 µM copper compared to the initial 1.25 mM copper in the sample.

EPR data were collected on a Bruker ELEXSYS E580 X- and Q-Band EPR Spectrometer (Billerica, MA, USA). Attenuation was adjusted to 11 dB (11 mW), modulation amplitude to 7.2 G, receiver gain to 60 dB, time constant to 40.90, conversion time 40.90, sweep time 41.94, center field 2600 G and sweep width 5000 G, 10 scans.

EPR spectra were simulated using Easyspin v. 5.2.35 and MATLAB R2021a. The pepper function of EasySpin was used to fit all spectra obtained in water/glycerol mixtures at 5 K.

## Figures and Tables

**Table 1:** Features of the EPR spectra of BSA-Cu(II)_NTS_ and BSA-Cu(II)_MBS_. The values were obtained by EasySpin simulation of the experimental spectra using the pepper function and varying g tensor, hyperfine coupling and linewidth peak to peak to achieve a high-quality fit (Figure S1).

	g Tensor	Hyperfine Coupling (MHz)
NTS	2.03, 2.17	8.44, 612
MBS	2.04, 2.05, 2.27	40, 12, 495

## Data Availability

The original contributions presented in this study are included in the article/supplementary material. Further inquiries can be directed to the corresponding authors.

## Supplementary Materials

The following supporting information can be downloaded at: https://www.mdpi.com/article/10.3390/molecules30020320/s1, Figure S1: X-band EPR spectra of BSA-Cu(II)_NTS_ (a) or BSA-Cu(II)_MBS_ (b); Figure S2: Peisach–Blumberg correlation diagram [38]; Figure S3: Fluorescence emission spectra of BSA (2 μM) titrated with Cu(II); Figure S4: X-band DPR spectra of Cu(II) in MOPS buffer (blue line) or in MOPS buffer with excess histidine (red line).
