# Supplementary figures and images for: Green Tea Polyphenol Epigallocatechin Gallate Interactions with Copper-Serum Albumin
Source: Molecules. 2025 Jan 15;30(2):320. doi: 10.3390/molecules30020320 (PMC11767587; doi:10.3390/molecules30020320)

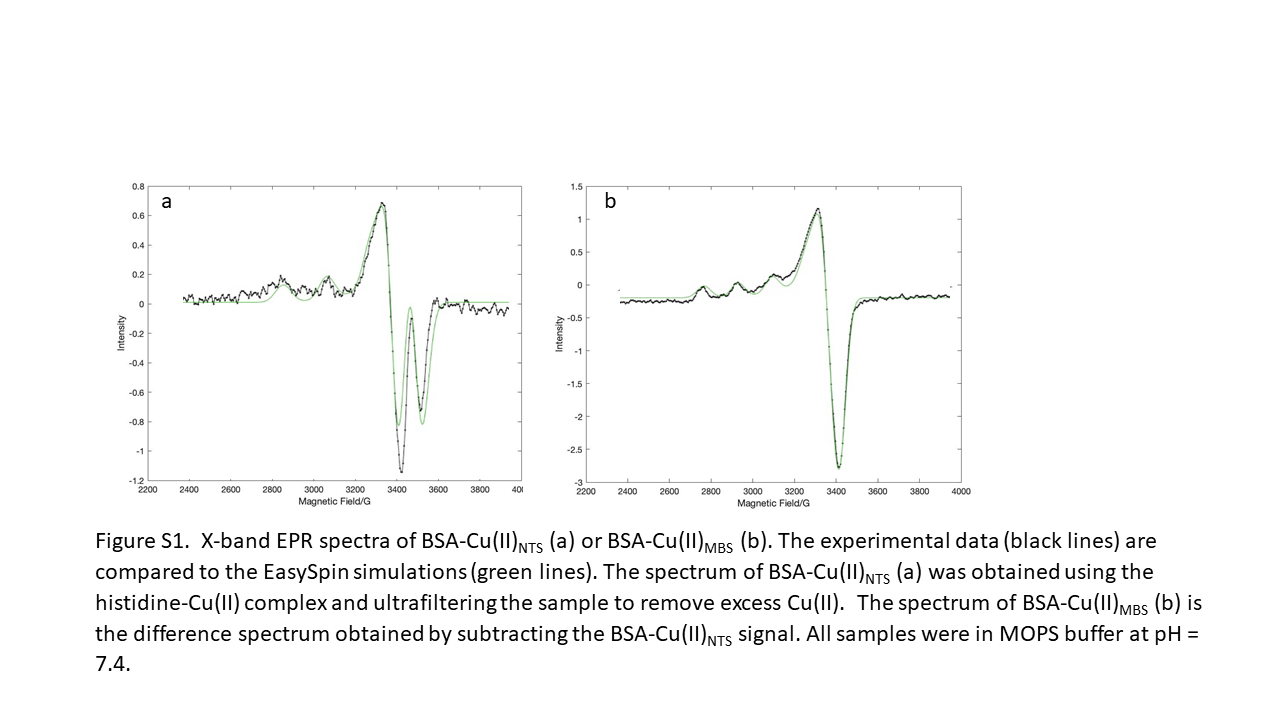

Supplement: Supplementary file 1 [file molecules-30-00320-s001.zip › Figure S1.tif]

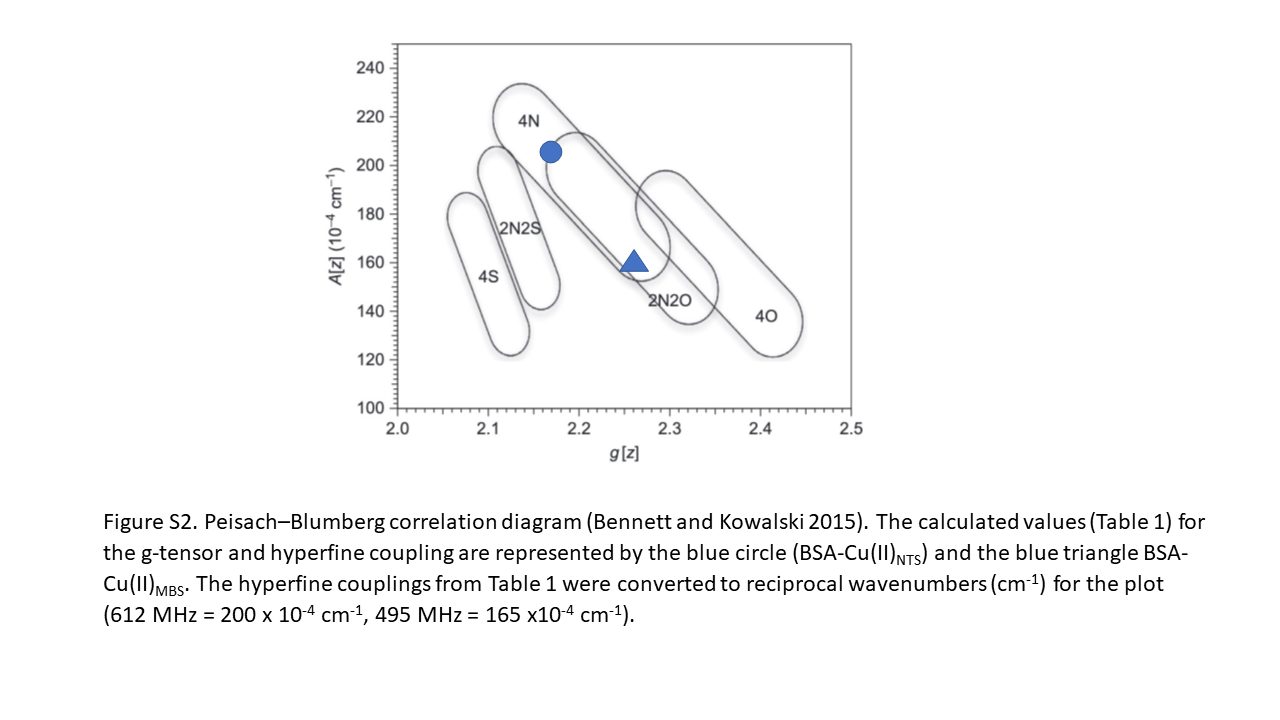

Supplement: Supplementary file 1 [file molecules-30-00320-s001.zip › Figure S2.tif]

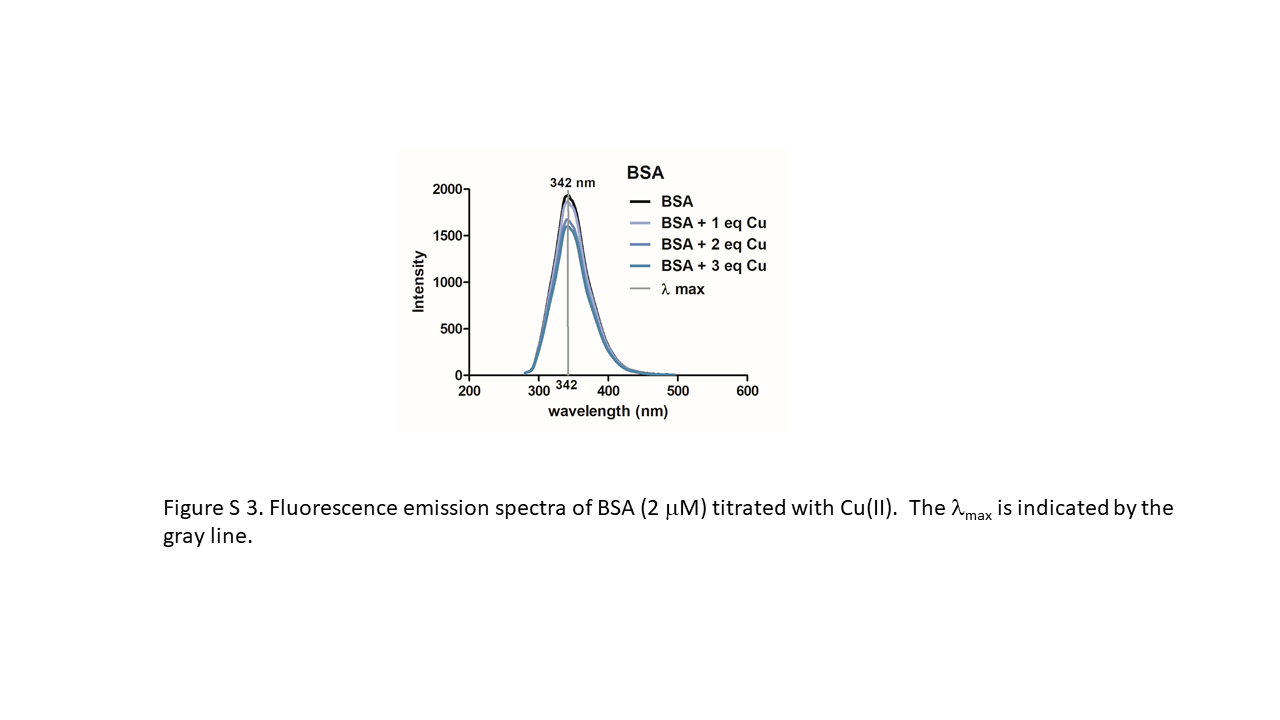

Supplement: Supplementary file 1 [file molecules-30-00320-s001.zip › Figure S3.tif]

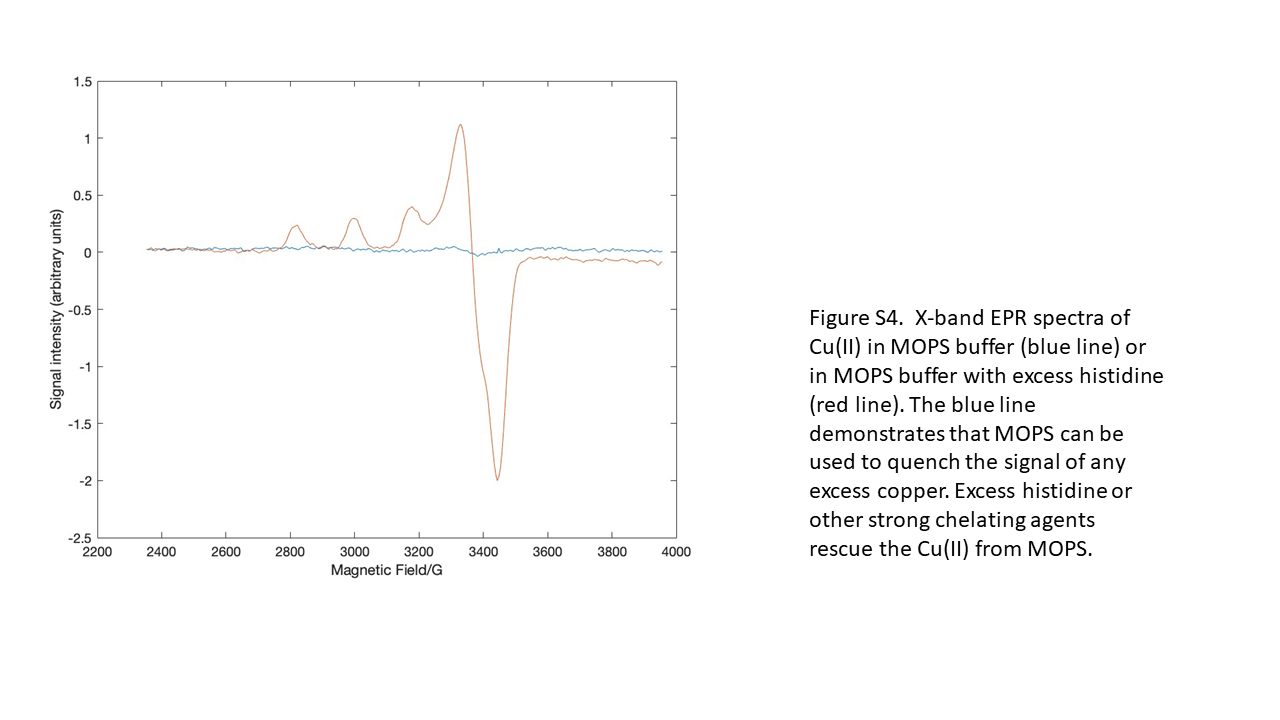

Supplement: Supplementary file 1 [file molecules-30-00320-s001.zip › Figure S4.tif]
